# Sensitivity of EQ-5D-3L, HUI2, HUI3, and SF-6D to changes in speech reception and tinnitus associated with cochlear implantation

**DOI:** 10.1007/s11136-018-2070-6

**Published:** 2018-11-27

**Authors:** A. Q. Summerfield, G. R. Barton, the UK Cochlear Implant Study Group

**Affiliations:** 1 Department of Psychology, University of York, York, YO10 5DD UK; 2 Hull York Medical School, University of York, York, YO10 5DD UK; 3 Norwich Medical School, University of East Anglia, Norwich, NR4 7TJ UK

**Keywords:** Preference-based measures, Health-related quality of life, Cochlear implantation, EQ-5D, HUI2/3, SF-6D

## Abstract

**Purpose:**

There is concern that some generic preference-based measures (GPMs) of health-related quality of life may be insensitive to interventions that improve hearing. Establishing where sensitivity arises could contribute to the design of improved measures. Accordingly, we compared the sensitivity of four widely used GPMs to a clinically effective treatment—cochlear implantation—which restores material degrees of hearing to adults with little or no functional hearing.

**Methods:**

Participants (*N* = 147) received implants in any of 13 hospitals in the UK. One month before implantation and 9 months after, they completed the HUI2, HUI3, EQ5D3L, and SF-6D questionnaires, together with the EuroQoL visual-analogue scale as a direct measure of health, a performance test of speech reception, and a self-report measure of annoyance due to tinnitus.

**Results:**

Implantation was associated with a large improvement in speech reception and a small improvement in tinnitus. HUI2 and HUI3 were sensitive to the improvement in speech reception through their *Sensation* and *Hearing* dimensions; EQ5D3L was sensitive to the improvement in tinnitus through its *Anxiety*/*Depression* dimension; SF-6D was sensitive to neither. Participants reported no overall improvement in health. Variation in health was associated with variation in tinnitus, not variation in speech reception.

**Conclusions:**

None of the four GPMs was sensitive to the improvements in both speech reception and tinnitus that were associated with cochlear implantation. To capture fully the benefits of interventions for auditory disorders, developments of current GPMs would need to be sensitive to both the health-related and non-health-related aspects of auditory dysfunction.

**Electronic supplementary material:**

The online version of this article (10.1007/s11136-018-2070-6) contains supplementary material, which is available to authorised users.

## Introduction

Generic preference-based measures (GPMs) of health-related quality of life (HRQL) play important roles in the allocation of resources in health care because they provide the utility component of cost–utility analyses. Such analyses inform prioritisation of treatments by third-party payers in many jurisdictions and are mandated in England when the National Institute for Health and Care Excellence (NICE) commissions the appraisal of a health technology [1].

There is concern that some GPMs are insensitive to interventions which improve hearing [2–5]. The issue is relevant because impaired hearing is prevalent. A clinically significant loss is experienced by 10% of all adults and by 30% of those older than 70 years [6, 7]. Sufferers must compete for resources to obtain treatments to alleviate their disability. There would be a failure of equity if treatments were denied not because they were ineffective but because GPMs failed to attribute appropriate value to their benefits.

The development of GPMs begins with researchers identifying dimensions on which good function corresponds to good health. Discrete levels of function are defined on each dimension ranging from poor to good. A subset of the possible combinations of levels is valued by a representative sample of the public. These informants use methods for measuring preferences [8] such that their valuations lie on a scale where 1 corresponds to perfect health, 0 to the state of being dead, and negative values to states considered worse than dead, if relevant. Statistical modelling is used to generate a valuation function which converts any combination of levels into a composite index whose value best aligns with the valuations of the informants. Finally, a questionnaire is compiled to elicit a respondent’s own level of function on each dimension. Then, the responses to the questionnaire and the valuation function are deployed to assign a value to the respondent’s HRQL that reflects public preferences.

We examined four widely used GPMs [9–11]. The EuroQol Descriptive System [9] includes dimensions relating to *Mobility, Self-care, Usual Activities, Pain*/*Discomfort*, and *Anxiety*/*Depression*. The version of the system until recently preferred by NICE [12] defines three levels on each dimension (EQ-5D-3L). The Mark-2 version of the Health Utilities Index (HUI2) [10, 13] includes dimensions relating to *Sensation, Mobility, Emotion, Cognition, Self-care*, and *Pain*, each with 4–6 levels. In the Mark-3 version (HUI3) [14], *Sensation* was decomposed into *Seeing, Hearing*, and (being understood when) *Speaking. Self-care* was re-worked as *Dexterity*. The Short-Form-6D (SF-6D) [11] includes dimensions relating to *Physical functioning, Role limitations, Social functioning, Pain, Mental health*, and *Vitality*, each with 4–6 levels. The dimensions are a subset of those in the Medical Outcomes Study Short-form Health Survey (SF-36) [15], such that an SF-6D utility can be derived from responses to the SF-36 [11].

Given differences in wording, dimensions, and methods of valuation, it is not surprising that the GPMs differ in their sensitivity to interventions [16], including interventions which improve hearing [e.g. 2–4]. A systematic review of studies that measured HRQL in participants with impaired hearing using one or more of EQ-5D, HUI3, and SF-6D [5] reached five conclusions: HUI3 was appropriate for estimating HRQL in studies involving impaired hearing; EQ-5D-3L was not responsive to modest changes in hearing; few studies had been designed to compare GPMs; only one study had included SF-6D; additional ‘head-to-head’ comparisons of GPMs were needed to understand why differences in sensitivity arose.

We made such a comparison by investigating the sensitivity of the four GPMs to cochlear implantation—a clinically effective intervention [17] which restores auditory sensations to people with little or no functional hearing [18, 19]. The primary goal of implantation is to improve the ability to understand speech. A secondary goal is to attenuate tinnitus [20, 21]—“the conscious experience of a sound that originates in an involuntary manner in the head of its owner, or may appear to do so” [22]. Up to 80% of candidates for implantation report some degree of tinnitus [23].

The rationale for the study was that the categorical change from dysfunctional hearing to viable hearing that is brought about by implantation would allow scope for GPMs to show improvement if they were sensitive to hearing. The study adds to those in the systematic review [5] of which only two were prospective evaluations of implantation where participants completed questionnaires themselves; both of those studies included only one GPM.

Against that background, we identified reasons why the four GPMs differed in their sensitivity to implantation. We distinguished their response to impaired speech reception from their response to tinnitus, and we considered the implications for the choice of GPM to use in studies of hearing.

## Methods

### Participants

Participants were adults who met criteria of candidacy for implantation in the United Kingdom (UK): they had developed a severe-to-profound sensorineural hearing loss in both ears after acquiring spoken language; they had at least one patent cochlear nerve; they could identify no more than 50% of the content words in pre-recorded sentences presented in quiet without lipreading when using hearing aids. Their demographic and audiological characteristics are listed in Table 1. They received an implant in one ear in any of 13 hospitals in the UK between June 1997 and May 2000. They were tested one month before implantation and again three and nine months after implantation as part of a larger study [18, 24, 25]. Results are reported for 147 participants (78 F, 69 M) who provided a complete set of outcome measures at the pre-operative and 9 month post-operative stages. Online Resource 1 provides evidence that these participants were representative of adults undergoing implantation in the UK.

**Table 1:** Biographical and audiological characteristics of participants before implantation

Measure	Minimum	Maximum	Mean	Standard deviation
Age at the time of implantation (years)	18.0	79.3	50.7	14.7
Pre-operative hearing level^a^ of the implanted ear (dB)	88.8	140.0	118.3	10.5
Pre-operative hearing leve^a^l of the other ear (dB)	93.8	140.0	117.5	10.5
Duration of severe-profound deafness^b^ of the implanted ear (years)	0.0	71.0	14.8	15.7
Duration of severe-profound deafness^b^ of the other ear (years)	0.0	71.0	14.9	15.4
Pre-operative speech-reception score (% correct)^c^	0.0	50.0	3.8	9.3

^a^Elevation of detection thresholds for pure tones averaged across the acoustic frequencies of 500, 1000, 2000, and 4000 Hz relative to normal hearing

^b^Self-reported by participants

^c^BKB Sentence Test [18, 29]

### Outcome measures

Five measures of HRQL and two functional measures of hearing were derived from four questionnaires and a performance test. The questionnaires were presented on touch screens. The performance test was conducted in audiological test rooms. The measures are described below where the name given to each derived variable is italicised, e.g. *EQ-5D-3L*.

#### Measures of HRQL

The EQ-5D-3L questionnaire and visual-analogue scale of the EuroQoL Descriptive System were presented. Reported levels of the five dimensions were converted to a composite index (*EQ-5D-3L*) using the valuation function described by Dolan [26]. The visual-analogue scale yielded a value in the range from zero (worst imaginable health) to 100 (best imaginable health) (*EQ-VAS*). For the Health Utilities Index Mark 2 and 3, a single questionnaire allowed both indices to be calculated. The words “hearing aid” were replaced with “cochlear implant” in the version of the questionnaire presented post-operatively. Reported levels of the dimensions were converted to weights which were combined to yield composite indices for HUI2 [13] and HUI3 [14] (*HUI2, HUI3*). For the Short-Form-6D, responses to the UK SF-36 questionnaire [27] were analysed to determine the level of function of each participant on each dimension and then to compute a composite index [28] *(SF-6D)*.

#### Functional measures of hearing

A measure of speech reception was obtained by presenting recordings of sentences to participants at an intensity typical of conversational speech and counting the percentage of content words reported correctly [18, 29] (*Speech*). A measure of annoyance due to tinnitus was obtained by presenting 13 questions [30; Online Resource 2] which probed the subjective manifestations of tinnitus and its psychological impact. Participants responded by positioning a pointer on a visual-analogue scale whose ends were labelled appropriately for each question. Responses were averaged and expressed as a number in the range from 0 (no annoyance) to 100 (great annoyance) (*Tinnitus*).

A good outcome from implantation is characterised by a high value of *Speech* and a low value of *Tinnitus*. Thus, to the extent that speech reception and annoyance due to tinnitus are associated with HRQL, *Speech* and *Tinnitus* are expected to display complementary patterns of correlation; where *Speech* correlates positively, *Tinnitus* should correlate negatively, and vice versa.

### Analyses

Analyses were conducted with IBM SPSS Statistics v.24 [31].

#### Sensitivity of outcome measures to implantation

We tested the sensitivity of each outcome measure to implantation by determining whether the change from the pre-operative to the post-operative value of the measure exceeded zero using a t test. Effect sizes (ES) were assessed with the Standardised Response Mean [32] and, for descriptive convenience, were classified as small (0.2 ≤ ES < 0.5), moderate (0.5 ≤ ES < 0.8), or large (0.8 ≤ ES). The changes in the four composite indices were compared in an analysis of variance. Mauchly’s test of sphericity was significant, so the Greenhouse-Geisser adjustment was made to the degrees of freedom.

#### Sensitivity of individual dimensions of GPMs to implantation

To establish which dimensions of each GPM were sensitive to implantation, and to determine whether positive changes on some dimensions were offset by negative changes on others, we identified dimensions on which there was a significant change—either positive or negative—between the levels to which participants assigned themselves before and after implantation using a Wilcoxon signed-rank test.

#### Sensitivity of composite indices to speech reception and tinnitus

A composite index could be sensitive to variation in both speech reception and tinnitus, to one, or to neither. To determine which pattern of sensitivity was shown by each GPM, we calculated Kendall rank correlation coefficients between changes in *Speech* and *Tinnitus* and changes in each composite index. We also examined whether values of *Speech* and *Tinnitus* before and, separately, after implantation were correlated with the pre- and post-operative values of the composite indices.

#### Sensitivity of individual dimensions to speech reception and tinnitus

To determine which individual dimensions underpinned correlations between composite indices and *Speech* and *Tinnitus*, we calculated Kendall rank correlation coefficients between changes in the levels of each dimension and changes in *Speech* and *Tinnitus*. We also tested whether values of *Speech* and *Tinnitus* before and after implantation were correlated with the pre- and post-operative levels of individual dimensions.

## Results

### Sensitivity of outcome measures to implantation

Table 2 reports the sensitivity of the outcome measures to implantation. In terms of effect size, *HUI2* displayed a moderate significant change and *HUI3* a large significant change. *EQ-5D-3L* displayed a small significant change. *SF-6D* and *EQ-VAS* did not show significant changes. Analysis of variance showed that the four changes differed significantly (F_(2.4,345.2)_ = 45.3, *p* < 0.001); all pair-wise comparisons were significant (*p* < 0.001), except the comparison between *EQ-5D-3L* and *SF-6D*.

**Table 2:** Comparison of values of outcome measures before and after implantation

	Outcome measure						
	*EQ-5D-3L*	*HUI2*	*HUI3*	*SF-6D*	*EQ-VAS*	*Speech*	*Tinnitus*
Mean pre-operative value	0.788	0.640	0.433	0.763	76.66	3.81	23.94
Mean post-operative value	0.827	0.775	0.629	0.775	77.93	56.08	16.81
Mean change in value^a^	0.040	0.135	0.197	0.012	1.26	52.27	− 7.13
Student’s *t*_146_^b^	2.30*	8.16***	12.10***	1.20	1.00	18.36***	− 4.05***
Adjusted SRM^c^	0.20	0.71	1.06	0.12	0.09	1.18	− 0.35

^a^Calculated as the post-operative value minus the pre-operative value. Improvements are shown by a negative change in *Tinnitus* (i.e. a reduction in annoyance) and by a positive change in other outcome measures

^b^*p < 0.05, ***p < 0.001

^c^Adjusted standardised response mean [32]

The first functional measure of hearing, *Speech*, increased from 3.8% correct to 56.1% correct with a large effect size. The second functional measure, *Tinnitus*, declined from 23.9 to 16.8 with a small effect size. Thus, implantation was associated with a large primary effect of improved speech reception and a small secondary effect of reduced annoyance due to tinnitus. Online Resource 3 provides additional support for the interpretation of speech reception as the primary outcome and annoyance due to tinnitus as the secondary outcome.

### Sensitivity of individual dimensions of GPMs to cochlear implantation

Table 3 summarises the sensitivity of the individual dimensions of the GPMs to implantation. For each dimension, the table includes the number of participants who placed themselves at each level before and after implantation, together with the results of a comparison of the 147 pairs of levels. As an example, consider the entry for the *Anxiety*/*Depression* dimension of EQ-5D-3L which is at top-right in the table. Before implantation, 83 participants placed themselves at the best level of this dimension, 52 at the middle level, and 12 at the worst level, summarised as (83,52,12). After implantation, the pattern had improved significantly to (113,30,4) (Wilcoxon *z* = 4.812, *p* < 0.001).

**Table 3:** Comparison of numbers of participants at each level of each dimension of GPMs before and after implantation

	EQ-5D-3L dimensions				
	Mobility	Self-care	Usual activities	Pain/discomfort	Anxiety/depression
N Pre^a^	(110,37,0)	(140,7,0)	(113,28,6)	(99,42,6)	(83,52,12)
N Post^b^	(108,39,0)	(139,8,0)	(118,27,2)	(89,52,6)	(113,30,4)
*Z* ^c,d^	− 0.47	− 0.33	+ 1.50	− 1.54	+ 4.81***

	HUI2 dimensions					
	Sensation	Mobility	Emotion	Cognition	Self-care	Pain
N Pre	(0,9,65,73)	(125,8,13,1,0)	(95,42,8,2,0)	(103,42,2,0)	(146,1,0,0)	(116,10,9,4,8)
N Post	(0,80,62,5)	(127,6,12,2,0)	(105,37,4,1,0)	(110,34,3,0)	(143,2,0,2)	(93,30,7,0,17)
*Z*	+ 8.92***	0.000	+ 1.79	+ 0.93	− 1.52	− 2.85**

	HUI3 dimensions							
	Vision	Hearing	Speaking	Ambulation	Dexterity	Emotion	Cognition	Pain
N Pre	(48,96,1,1,1,0)	(3,1,11,4,56,72)	(106,21,13,5,2)	(125,8,13,0,1,0)	(145,0,0,2,0,0)	(97,37,7,5,1)	(103,12,16,13,3,0)	(85,27,24,8,3)
N Post	(41,104,0,1,1,0)	(0,2,107,0,33,5)	(114,22,11,0,0)	(127,6,12,1,0,1)	(147,0,0,0,0,0)	(94,41,12,0,0)	(110,7,14,12,4,0)	(72,37,27,10,1)
Z	− 1.26	+ 9.22***	+ 2.08*	+ 0.17	+ 1.41	+ 0.30	+ 0.64	− 1.28

	SF-6D dimensions					
	Physical	Role limitations	Social	Pain	Mental health	Vitality
N Pre	(56,46,19,7,15,4)	(95,19,16,17)	(96,16,22,9,4)	(73,22,29,9,11,3)	(27,46,45,26,3)	(13,75,40,13,6)
N Post	(51,43,29,7,13,4)	(96,10,14,27)	(93,21,24,6,3)	(60,30,21,22,12,2)	(51,42,40,12,2)	(17,70,45,11,4)
*Z*	− 0.80	− 1.11	+ 0.24	− 2.13*	+ 4.53***	+ 0.86

^a^*N Pre* Number of participants at each level of a dimension before implantation. Left-hand number in parentheses is the number at the most advantageous level of the dimension; right-hand number is the number at the least advantageous level

^b^*N Post* Number of participants at each level of a dimension after implantation

^c^*Z* statistic from Wilcoxon signed-rank comparison of pre- and post-operative levels. A negative value indicates that the mean level became less advantageous. A positive value indicates that the mean level became more advantageous

^d^*p < 0.05, **p < 0.01, ***p < 0.001

Implantation was associated with three types of change: improvements in *Sensation* (HUI2), *Hearing* (HUI3), and *Speaking* (HUI3); improvements in *Anxiety*/*Depression* (EQ-5D-3L) and *Mental Health* (SF-6D); and a worsening of *Pain* (HUI2, SF-6D). Thus, each GPM included at least one dimension that displayed a significant improvement, while HUI2 and SF-6D also included dimensions that worsened significantly.

### Sensitivity of measures of HRQL to speech reception and tinnitus

The upper panel of Table 4 lists Kendall rank-order correlation coefficients between change in *Speech* and *Tinnitus* and change in each measure of HRQL. Change in *Speech* was correlated with change in *HUI2* and *HUI3*, but not with change in *EQ-5D-3L* or *EQ-VAS*. Change in *Tinnitus* showed the opposite pattern; it was correlated with change in *EQ-5D-3L* and *EQ-VAS*, but not with change in *HUI2* or *HUI3*. Change in neither functional measure was correlated with change in *SF-6D*. Thus, none of the composite indices was sensitive to the improvements in both speech reception and tinnitus associated with implantation. Online Resource 4 includes multiple regression analyses which corroborate these patterns of correlation.

**Table 4:** Kendall rank-order coefficients of correlation among functional measures of hearing and measures of HRQL

	*Tinnitus*	*EQ-5D-3L*	*HUI2*	*HUI3*	*SF-6D*	*EQ-VAS*
Change in values^a^						
*Speech*	− 0.056	0.118	0.185**	0.191**	0.051	− 0.004
*Tinnitus*		− 0.182*	− 0.112	− 0.069	− 0.054	− 0.179**
Pre-operative values^b^						
*Speech*	− 0.053	0.022	0.178 *	0.137	0.135	0.053
*Tinnitus*		− 0.223**	− 0.230**	− 0.242**	− 0.247**	− 0.225**
Post-operative values^c^						
*Speech*	0.002	0.128	0.195**	0.189**	0.136	0.044
*Tinnitus*		− 0.242**	− 0.201**	− 0.206**	− 0.251**	− 0.236**

Low values of *Tinnitus*, but high values of *Speech* and of measures of HRQL, were advantageous pre- and post-operatively. A negative change in *Tinnitus*, but a positive change in *Speech* and in measures of HRQL, represented improvements. Thus, correlations between *Tinnitus* and measures of HRQL were negative, while correlations between *Speech* and measures of HRQL were positive

Significance assessed against Bonferroni-adjusted alpha levels of 0.01 per test (0.05/5) (*) and 0.002 per test (0.01/5) (**)

^a^Correlations among changes in measures; change was calculated as the post-operative value minus the pre-operative value

^b^Correlations among pre-operative values of measures

^c^Correlations among post-operative values of measures

The middle and lower panels of Table 4 list coefficients of correlation between the functional measures of hearing and the measures of HRQL before and after implantation. Of the five measures of HRQL, only *HUI2* was correlated with *Speech* before implantation, and only *HUI2* and *HUI3* were correlated with *Speech* after implantation. In contrast, each measure of HRQL was correlated with *Tinnitus*, both before and after implantation.

### Sensitivity of individual dimensions to speech reception and tinnitus

Table 5 lists coefficients of correlation between the levels of individual dimensions and measures of *Speech* and *Tinnitus*. The results corroborate the implication of Table 4 that the GPMs were more attuned to variation among participants in *Tinnitus* than in *Speech*. The four GPMS together contain 25 dimensions. Variation in *Speech* was correlated significantly with the levels of only four of them before or after implantation, while variation in *Tinnitus* was correlated with 15.

**Table 5:** Kendall rank-order coefficients of correlation^a^ between functional measures of hearing and levels of individual dimensions of GPMs

GPM	Dimension	Pre-operative		Post-operative		Change	
		*τ* ^b^	Sig.^e^	*τ* ^c^	Sig.^e^	*τ* ^d^	Sig.^e^
(a) Correlations with *Speech*							
EQ-5D-3L	*Mobility*	− 0.090		− 0.041		0.020	
	*Self-care*	0.030		− 0.097		− 0.015	
	*Usual activities*	0.036		− 0.019		− 0.060	
	*Pain*/*Discomfort*	− 0.021		− 0.064		− 0.003	
	*Anxiety*/*Depression*	0.020		− 0.212	*	− 0.174	*
HUI2	*Sensation*	− 0.359	**	− 0.263	**	− 0.230	**
	*Mobility*	− 0.112		− 0.083		− 0.096	
	*Emotion*	− 0.008		− 0.068		− 0.082	
	*Cognition*	0.094		− 0.154		− 0.054	
	*Self-care*	− 0.047		− 0.027		− 0.057	
	*Pain*	0.052		− 0.075		− 0.040	
HUI3	*Vision*	0.055		− 0.071		− 0.058	
	*Hearing*	− 0.325	**	− 0.193	*	− 0.225	**
	*Speaking*	0.074		− 0.165		0.023	
	*Ambulation*	− 0.112		− 0.083		− 0.096	
	*Dexterity*	− 0.066		^f^		− 0.071	
	*Emotion*	− 0.022		− 0.061		− 0.071	
	*Cognition*	0.092		− 0.159		− 0.085	
	*Pain*	− 0.060		− 0.102		0.006	
SF-6D	*Physical functioning*	− 0.098		− 0.112		− 0.097	
	*Role limitations*	− 0.092		− 0.078		− 0.019	
	*Social functioning*	− 0.131		− 0.115		− 0.134	
	*Pain*	− 0.051		− 0.090		− 0.047	
	*Mental Health*	− 0.163		− 0.201	**	− 0.055	
	*Vitality*	− 0.035		− 0.025		− 0.031	
(b) Correlations with *Tinnitus*							
EQ-5D-3L	Mobility	0.216	*	0.227	**	0.062	
	*Self-care*	0.072		− 0.017		− 0.031	
	*Usual activities*	0.204	*	0.250	**	0.150	
	*Pain*/*discomfort*	0.180	*	0.208	*	0.134	
	*Anxiety*/*depression*	0.136		0.194	*	0.185	*
HUI2	*Sensation*	0.207	*	0.054		0.042	
	*Mobility*	0.173		0.123		− 0.103	
	*Emotion*	0.076		0.125		0.053	
	*Cognition*	0.174		0.171		0.120	
	*Self-care*	0.007		− 0.033		− 0.126	
	*Pain*	0.137		0.201	*	− 0.004	
HUI3	*Vision*	− 0.095		0.073		− 0.029	
	*Hearing*	0.193	*	0.083		0.043	
	*Speaking*	0.041		0.003		− 0.032	
	*Ambulation*	0.173		0.123		− 0.103	
	*Dexterity*	0.068		^f^		− 0.075	
	*Emotion*	0.199	*	0.197	*	0.034	
	*Cognition*	0.181	*	0.167		0.124	
	*Pain*	0.162		0.189	*	0.050	
SF-6D	*Physical functioning*	0.164		0.149		− 0.012	
	*Role limitations*	0.172	*	0.255	**	0.133	
	*Social functioning*	0.245	**	0.178	*	0.142	
	*Bodily pain*	0.167		0.212	**	0.053	
	*Mental health*	0.132		0.195	*	0.102	
	*Vitality*	0.192	*	0.270	**	0.116	

^a^Levels of dimensions were quantified with sequential integers. The value 1 was assigned to the most advantageous level. Thus, low values of level were advantageous pre- and post-operatively, and a reduction in level represented an improvement. Low values of *Tinnitus*, but high values of *Speech* and of measures of HRQL, were advantageous pre- and post-operatively. A negative change in *Tinnitus*, but a positive change in *Speech* and in measures of HRQL, represented improvements. Therefore, correlations between *Tinnitus* and level were generally positive, while correlations between *Speech* and level were generally negative

^b^Kendall rank-order coefficients of correlation between pre-operative values of measures

^c^Kendall rank-order coefficients of correlation between post-operative values of measures

^d^Kendall rank-order coefficients of correlation between changes in measures calculated as the post-operative value minus the pre-operative value

^e^Significance assessed against Bonferroni-adjusted alpha levels of 0.05/n (*) and 0.01/n (**), where n is the number of dimensions in the GPM (5 for EQ-5D-3L, 6 for HUI2, 8 for HUI3, and 6 for SF-6D).

^f^Could not be calculated because all participants placed themselves at the most advantageous level of the *Dexterity* dimension after implantation

## Discussion

The data reported here were gathered in the late 1990s. Improvements in cochlear implants and changes in candidature might have rendered the results irrelevant to today’s outcomes. Two considerations counter those concerns. First, criteria of candidacy for unilateral implantation of adults in England [17] remain the same as the entry criteria for the study, although they are currently under review [33]. Second, accuracy of speech reception following implantation has plateaued since the mid-1990s [34]. Thus, participants in the study, and patients receiving implants in England today, are likely to come from the same population and to demonstrate the same patterns of association among outcome measures.

With those caveats, the study provides evidence at three levels of detail to inform the choice of GPM to use in assessments of interventions for hearing disorders. First, at the level of composite indices, the results corroborate previous demonstrations [2–5] that GPMs differ in their sensitivity to treatments which improve hearing (Table 2): HUI2 and HUI3 showed moderate or large responses to implantation; EQ-5D-3L showed a small response; and SF-6D showed no response. In consequence, economic analyses informed by HUI2 or HUI3 suggest that implantation is a cost-effective intervention, while analyses informed by EQ-5D-3L or SF-6D suggest that it is not (Online Resource 5). That result might argue that only HUI3 need be used in studies of hearing.

However, limitations in all of the GPMs emerge at the second level of detail where the changes associated with implantation on individual dimensions are considered (Table 3). Significant changes occurred on three groups of dimensions: positive changes resulting from improved auditory sensitivity; positive changes resulting from improved psychological well-being; and negative changes presumably resulting from pain caused by the surgical wound and from irritation induced by wearing the external parts of the implant system against the scalp. Limitations of the GPMs are shown by the fact that no GPM displayed all three types of change.

It is not surprising that the composite indices of HUI2 and HUI3 were sensitive to the improvement in speech reception, given that the questions which map respondents onto the levels of the *Sensation* (HUI2) and *Hearing* (HUI3) dimensions ask about the ability to “hear what is said” in conversation. More surprising may be that neither *Usual Activities* (EQ-5D-3L) nor *Role Limitations* or *Social Functioning* (SF-6D) were similarly sensitive. However, participants had been severely profoundly hearing impaired for nearly 15 years on average (Table 1), so their “usual” activities would have adapted to constraints imposed by their deafness. Also, the questions that map respondents onto levels of the *Role Limitations* and *Social Functioning* dimensions emphasise limitations arising from health. The loss of hearing sensitivity that underpins impaired speech reception is not usually the consequence of disease [35] nor is it generally perceived to be a manifestation of ill-health [36].

In contrast, evidence from the third level of detail—individual differences in outcome measures before and after implantation—shows that annoyance due to tinnitus was perceived to be strongly related to perceptions of health (Table 4b). It was associated with poorer levels of function on dimensions related to physical activity (*Mobility* and *Usual Activities* in EQ-5D-3L, *Role Limitations* and *Social Function* in SF-6D), pain (*Pain*/*Discomfort* in EQ-5D-3L, *Pain* in HUI2, HUI3, SF-6D), and mental health (*Anxiety*/*Depression* in EQ-5D-3L, *Emotion* in HUI3, *Mental Health* in SF-6D). This pattern (see also [37]) can be rationalised by the ideas that the negative influence of tinnitus on the capacity to concentrate reduces productive activity [38], that tinnitus is discomforting and akin to pain [39, 40], and that tinnitus either elevates anxiety [41] or anxiety exacerbates the experience of tinnitus [42].

The conclusion that the GPMs are more attuned to tinnitus than impaired speech reception is reinforced by the finding that variation among participants in the change in self-reported health, EQ-VAS, was correlated with the change in *Tinnitus*, not the change in *Speech* (Table 4). The conclusion is supported by supplementary analyses in Online Resource 6 and is also compatible with analyses reported by Konerding et al. [16] who examined patterns of association among dimensions of EQ-5D-3L, HUI2, and SF-6D. Correlated patterns across the three GPMs were found among dimensions related to mental health and, separately, physical functioning, and physical pain. The *Sensation* dimension of HUI2, however, was not related to other dimensions, suggesting that sensory deficits are independent of other aspects of health. That conclusion anticipates the demonstration in the present paper and elsewhere [2–5] that sensitivity to interventions which improve the ability to detect sounds is shown only by those GPMs that explicitly measure the benefits of improved auditory sensitivity.

Which GPM should be used in studies of hearing? Consider first that *Tinnitus* was associated with aspects of health which are represented by dimensions in each GPM. All of the GPMs, therefore, have the potential to be sensitive to changes in health produced by interventions which reduce annoyance due to tinnitus. Although in the present study only EQ-5D-3L was sensitive to the small improvement in *Tinnitus* (Table 4), in a clinical trial reported by Maes et al. [43], both EQ-5D-3L and HUI3 distinguished patients whose tinnitus improved from patients whose tinnitus did not improve, with HUI3 showing slightly greater sensitivity. Thus, in jurisdictions where policy makers prefer HUI3, it should be used in studies of hearing, insofar as it has been shown to be sensitive to improvements in both tinnitus and auditory sensitivity.

In jurisdictions where policy makers prefer EQ-5D, it should be used to evaluate interventions intended to improve tinnitus, while HUI3 should be used to evaluate interventions intended to improve hearing sensitivity. That distinction aligns with the guidance given by NICE that alternatives to EQ-5D should be used to evaluate conditions for which EQ-5D lacks critical dimensions of health [1]. Nonetheless, it is unsatisfactory to advocate the use of different GPMs to assess treatments for two aspects of dysfunctional hearing which can co-occur and which can both respond to the same treatment. The dilemma reinforces the aim of a consortium of researchers in the UK [44] to develop a successor to the EuroQol Descriptive System which preserves sensitivity to the conventional dimensions of health while adding sensitivity to sensory disorders [45, 46] among other changes.

## Electronic supplementary material

Below is the link to the electronic supplementary material.

Supplementary material 1 (PDF 971 KB)
