# Supplementary material for: Sensitivity of EQ-5D-3L, HUI2, HUI3, and SF-6D to changes in speech reception and tinnitus associated with cochlear implantation
Source: Qual Life Res. 2018 Nov 27;28(5):1145–54. doi: 10.1007/s11136-018-2070-6 (PMC6470108; doi:10.1007/s11136-018-2070-6)
Supplement: Supplementary file 1 — Supplementary material 1 (PDF 971 KB) [file 11136_2018_2070_MOESM1_ESM.pdf]

Online Resources (Electronic Supplementary Material)

Article title: Sensitivity of EQ-5D-3L, HUI2, HUI3, and SF-6D to changes in speech reception and tinnitus associated with cochlear implantation

Journal name: Quality of Life Research

Author names: A. Q. Summerfield & G. R. Barton

Corresponding author: Quentin Summerfield

Affiliation of the corresponding author: Department of Psychology, University of York, York, YO10 5DD, UK

E-mail address: quentin.summerfield@york.ac.uk

This document contains the online resources associated with this article.

Online Resource	Title	Page
1	Representativeness of the sample of 147 participants	2
2	Assessment of annoyance due to tinnitus	7
3	Primary and secondary outcomes from implantation	8
4	Multiple regression analyses	11
5	Analyses of cost effectiveness	15
6	Relationship of *Speech* and *Tinnitus* to physical health and psycho-social well-being	23

Online Resource 1: Representativeness of the sample of 147 participants

The 147 participants in the present study are a sub-set of 316 participants who were recruited into a larger study reported in three papers by the UK Cochlear Implant Study Group in 2004 (UKCISG, 2004a,b,c). Participants in that study were invited to complete a battery of performance tests and questionnaires before implantation and then again 3 and 9 months after implantation. The battery included the performance test and questionnaires from which results are reported in the present paper.

Participants in the larger study were sequential cases referred for implantation to any one of 13 hospitals in the UK National Health Service who met seven inclusion criteria:

1. They consented to participate.
2. They had a severe or profound sensorineural hearing impairment, defined as an average of pure-tone thresholds at .5, 1, 2, and 4 kHz >70 dB in the better-hearing ear.
3. They were post-lingually deafened.
4. They were adults at the time of recruitment: ≥16 years of age (participants in Scotland) and ≥18 years of age (participants in England, Wales, and Northern Ireland).
5. They were able to identify no more than 50% of the content words correctly in a hi-fidelity pre-recorded test of the ability to perceive words in sentences without lipreading when using acoustic hearing aids.
6. They had not previously been in receipt of a cochlear implant.
7. They received a unilateral multi-channel implant between June 1st 1997 and May 31st 2000 and were scheduled to have completed a protocol of post-operative assessments by May 31st 2001.

Table 3 in UKCISG (2004a) explains that 316 participants were recruited. Of them, 311 received an implant and participated to at least some degree in the study. Of the 311, 147 met two further inclusion criteria:

8. They had received an implant by 31st December 2000.
9. They had provided complete set of outcome measures for the present study as described in Figure 1 in the present paper at the pre-operative and 9-month post-operative stages.

Due to an error in archiving, data were not available from potential additional participants who might have met Criterion 9 but who were implanted after 31st December 2000.

In what follows, we refer to the larger group of 311 participants as the '311-dataset' and the subset who were included in the present study as the '147-dataset'.

Tables OR1.1 and OR1.2 show that the members of the 147-dataset possessed similar biographical and audiological characteristics to the members of the 311-dataset. Table OR1.3 demonstrates that the mean values of the outcome measures (pre-implantation, post-implantation, and the difference between the two) were similar for the members of the 147-dataset and for those members of the 311-dataset who provided each outcome measure.

We judge that the 147 participants who provided complete sets of data are representative of the larger group of 311 patients. Additionally, insofar as the larger group was made up of sequential consenting referrals, we judge that the 147 participants are representative of adults who received unilateral cochlear implants in the UK in the late 1990's.

Table OR1.1: Biographical and audiological data recorded before implantation for members of the 147-dataset (black font) and the 311-dataset (*red italic font*).

Measure	Dataset	Minimum	Maximum	Median	Inter-quartile range	Mean	Standard deviation
Age at the time of implantation (years)	147	18.0	79.3	53.1	21.9	50.7	14.7
	311	*16.0*	*82.0*	*52.0*	*23.0*	*50.8*	*15.4*
Hearing level[a] of implanted ear (dB)	147	88.8	140.0	117.5	17.5	118.3	10.5
	311	*87.5*	*140.0*	*118.8*	*17.5*	*118.5*	*10.2*
Hearing level[a] of other ear (dB)	147	93.8	140.0	116.3	18.8	117.5	10.5
	311	*85.0*	*140.0*	*118.8*	*17.5*	*118.2*	*10.6*
Duration of severe-profound deafness[b] in the implanted ear (years)	147	0.0	71.0	8.2	15.9	14.8	15.7
	311	*0.0*	*71.0*	*8.2*	*15.7*	*13.8*	*14.5*
Duration of severe-profound deafness[b] in the other ear (years)	147	0.0	71.0	8.3	20.1	14.9	15.4
	311	*0.0*	*71.0*	*9.5*	*19.9*	*15.4*	*15.8*
Speech reception (% correct)[c]	147	0.0	50.0	0.0	2.0	3.8	9.3
	311	*0.0*	*50.0*	*0.0*	*2.0*	*3.6*	*8.7*

[a]Elevation of detection thresholds for pure tones averaged across the acoustic frequencies of .5, 1, 2, and 4 kHz relative to normal hearing.

[b]Self-reported by participants.

[c]BKB Sentence Test (Bench, Kowal, & Bamford , 1979; UKCISG, 2004a).

Table OR1.2: Age and gender profiles of the members of the 147-dataset (black font) and the 311-dataset (*red italic font*). For each age band, and each gender, the number (N) of participants is tabulated along with the percentage of the total number of participants in the dataset (% of total).

Age band (Years)	Dataset	Women N (% of total)	Men N (% of total)	Total N (% of total)
16-17	147	0 (0.0)	0 (0.0)	0 (0.0)
	311	*2 (0.6)*	*0 (0.0)*	*2 (0.6)*
18-19	147	1 (0.7)	2 (1.4)	3 (2.0)
	311	*2 (0.6)*	*2 (0.6)*	*4 (1,3)*
20-24	147	4 (2.7)	3 (2.0)	7 (4.8)
	311	*2 (0.6)*	*1 (0.3)*	*3 (1.0)*
25-29	147	4 (2.7)	4 (2.7)	8 (5.4)
	311	*7 (2.3)*	*8 (2.6)*	*15 (4.8)*
30-34	147	2 (1.4)	5 (3.4)	7 (4.8)
	311	*14 (4.5)*	*5 (1.6)*	*19 (6.1)*
35-39	147	6 (4.1)	4 (2.7)	10 (6.8)
	311	*10 (3.2)*	*5 (1.6)*	*15 (4.8)*
40-44	147	8 (5.4)	3 (2.0)	11 (7.5)
	311	*11 (3.5)*	*11 (3.5)*	*22 (7.1)*
45-49	147	12 (8.2)	9 (6.1)	21 (14.3)
	311	*16 (5.1)*	*10 (3.2)*	*26 (8.4)*
50-54	147	6 (4.1)	4 (2.7)	10 (6.8)
	311	*19 (6.1)*	*17 (5.5)*	*36 (11.6)*
55-59	147	13 (8.8)	13 (8.8)	26 (17.7)
	311	*24 (7.7)*	*13 (4.2)*	*37 (11.9)*
60-64	147	8 (5.4)	9 (6.1)	17 (11.6)
	311	*16 (5.1)*	*20 (6.4)*	*36 (11.6)*
65-69	147	6 (4.1)	9 (6.1)	15 (10.2)
	311	*13 (4.2)*	*26 (6.4)*	*39 (12.5)*
70-74	147	5 (3.4)	3 (2.0)	8 (5.4)
	311	*10 (3.2)*	*21 (6.8)*	*31 (10.0)*
75-79	147	3 (2.0)	1 (0.7)	4 (2.7)
	311	*7 (2.3)*	*6 (1.9)*	*13 (4.2)*
80-84	147	0 (0.0)	0 (0.0)	0 (0.0)
	311	*5 (1.6)*	*6 (1.9)*	*11 (3.5)*
85-89	147	0 (0.0)	0 (0.0)	0 (0.0)
	311	*1 (0.3)*	*1 (0.3)*	*2 (0.6)*
**16-89**	**147**	**78 (53.1)**	**69 (46.9)**	**147 (100.0)**
	311	***159 (51.1)***	***152 (48.9)***	***311 (100.0)***

Table OR1.3: Mean values of outcome measures for the members of the 147-dataset (black font) and the 311-dataset (*red italic font*). The number in parentheses below each mean for the 311-dataset is the number of participants who provided the outcome measure. For the 147-dataset, this value was always 147. The values of Change for the 311-dataset were calculated as the post-implantation mean minus the pre-implantation mean, ignoring the fact that different numbers of participants contributed to the pre- and post-implantation means. The GBI provides a direct measure of change. It was completed by 254 members of the 311-dataset.

	Dataset	*Speech*	*Tinnitus*	*EQ-5D-3L*	*EQ(vas)*	*HUI2*	*HUI3*	*SF-6D*	*GHSI*	*GBI*
Pre-implantation mean	147	3.81	23.94	.788	76.66	.640	.433	.763	40.14	-
	311	*3.62*	*27.0*	*.810*	*76.65*	*.636*	*.433*	*.755*	*40.89*	
	(N)	*(311)*	*(305)*	*(275)*	*(276)*	*(296)*	*(296)*	*(296)*	*(271)*	
Post-implantation mean	147	56.08	16.81	.827	77.93	.775	.629	.775	57.60	-
	311	*54.12*	*18.52*	*.843*	*79.06*	*.777*	*.638*	*.771*	*57.99*	
	(N)	*(295)*	*(263)*	*(212)*	*(212)*	*(223)*	*(223)*	*(279)*	*(241)*	
Change (Post-Pre)	147	52.27	-7.13	.040	1.26	.135	.197	.012	17.46	43.95
	311	*52.27*	*-9.37*	*.032*	*2.41*	*.141*	*.205*	*.016*	*17.10*	*44.78*
	(N)									*(254)*

References

Bench, J., Kowal, A., Bamford, J. (1979). The BKB (Bamford-Kowal-Bench) sentence lists for partially-hearing children. British Journal of Audiology 13: 108-112.

UK Cochlear Implant Study Group. (2004a). "Criteria of candidacy for unilateral cochlear implantation in postlingually deafened adults I: Theory and measures of effectiveness." *Ear and Hearing* 25: 310-335.

UK Cochlear Implant Study Group. (2004b). Criteria of candidacy for unilateral cochlear implantation in postlingually deafened adults II: Cost-effectiveness analysis. *Ear and Hearing* 25: 336-360.

UK Cochlear Implant Study Group. (2004c). Criteria of candidacy for unilateral cochlear implantation in postlingually deafened adults III: Prospective evaluation of an actuarial approach to defining a criterion. *Ear and Hearing* 25: 360-374.

Online Resource 2: Assessment of annoyance due to tinnitus

Table OR2.1 contains the wording of questions which contributed to the measure of annoyance due to tinnitus from Sissons (1996). Participants responded to each question by positioning a slider on a visual-analogue scale whose ends were labelled with the words in the third column of the table.

Table OR2.1: Questions contributing to the measure of annoyance due to tinnitus.

Number	Question	Labels at ends of visual-analogue scale
	DURING TIMES WHEN YOU ARE AWARE OF YOUR TINNITUS:	
1a	How often does your tinnitus make you feel IRRITABLE?	Never/Always
1b	How often does your tinnitus make you feel ANXIOUS?	Never/Always
1c	How often does your tinnitus make you feel UNHAPPY?	Never/Always
1d	How often does your tinnitus PREVENT YOU FROM RELAXING?	Never/Always
1e	How often does your tinnitus make you feel ANNOYED?	Never/Always
1f	How often does your tinnitus make you feel HELPLESS?	Never/Always
2	When you are aware of your tinnitus, how often does it PREVENT YOU CONCENTRATING, for example while trying to read?	Never/Always
3	When you have tinnitus, how much of the time is your ATTENTION drawn to it?	Not at all/All of the time
4	How much DISCOMFORT does your tinnitus cause you during a normal day?	No discomfort/A great deal of discomfort
5	How much does your tinnitus reduce the QUALITY OF YOUR LIFE overall?	Not at all/Makes life not worth living
6	How LOUD would you say your tinnitus is?	Extremely quiet/Extremely loud
7	HOW MUCH DO YOU AGREE with the statement: Tinnitus has made me feel less interested in GOING OUT.	Completely Disagree/Completely Agree
8	HOW MUCH DO YOU AGREE with the statement: Tinnitus contributes to a general feeling of ILL HEALTH.	Completely Disagree/Completely Agree

Reference

Sissons, C. (1996). *Tinnitus questionnaire items for measures of severity, maskability, and intrusion/annoyance.* Dissertation presented to the Department of Public Health and Epidemiology, University of Nottingham.

Online Resource 3: Primary and secondary outcomes from implantation

The judgement that improvement in speech reception is the primary aim of cochlear implantation, while reduction in annoyance due to tinnitus is a secondary benefit, reflects clinical intention. It was conceivable that the order was reversed in terms of the impact of the changes on participants. We tested that idea with four questions which measured aspects of satisfaction with implantation. Responses to the questions were made on 5-point Likert response scales. The questions and the labels given to the responses are included in Table OR3.1. The numbers of participants who made each response are listed in Table OR3.2.

Table OR3.1: Questions which explored satisfaction with implantation and the labels given to the 5-point Likert response scales.

Question Number	Question	Labels				
		Response 1	Response 2	Response 3	Response 4	Response 5
Q1	How successful do you think your cochlear implant is?	Great or moderate failure	Partial failure	No change	Partial success	Great or moderate success
Q2	Do you feel pleased or disappointed about getting a cochlear implant?	Greatly or moderately disappointed	A little or somewhat disappointed	No change	A little or somewhat pleased	Greatly or moderately pleased
Q3	How successful do members of your family and close friends think your cochlear implant is?	Great or moderate failure	Partial failure	No change	Partial success	Great or moderate success
Q4	If you knew that someone else in your family or a close friend had a similar condition to yours, would you encourage them to get a similar cochlear implant?	Definitely not	Probably not	Can't decide	Probably yes	Definitely yes

Table OR3.2: Numbers of participants out of 147 who made each response.

Question Number	Response 1	Response 2	Response 3	Response 4	Response 5
Q1	1	1	1	16	128
Q2	3	2	1	7	134
Q3	1	3	3	14	126
Q4	0	0	7	17	123

We conducted binary logistic regression analyses to determine the extent to which changes in *Speech* and *Tinnitus* were predictors of whether participants gave the most favourable response to a question or any other response. To allow the influence of the two predictors to be compared, we transformed their values in two ways. First, we multiplied the value of the change in *Tinnitus* by minus one to create the variable *Negative Tinnitus.* As a result, positive changes in both predictors would be expected to be associated with better outcomes. Second, we standardised the values of change in *Speech* and change in *Negative Tinnitus* so that their relative influence could be assessed by comparing the size of their β-coefficients.

The results of these analyses are plotted in Figure OR3.1 where the heights of the grey vertical bars plot the size of the β-coefficients for the standardised change in *Speech* and the heights of the white vertical bars plot the β-coefficients for the standardised change in *Negative Tinnitus*. Error bars plot BCa 95% confidence intervals. Changes in both *Speech* and *Negative Tinnitus* were significant predictors of the response to Question 1 (How successful do you think your cochlear implant is?). Neither was a significant predictor of the response to Question 2 (Do you feel pleased or disappointed about getting a cochlear implant?) where the smallest number of participants gave a response other than the most favourable response. Change in *Speech,* but not change in *Negative Tinnitus*, was a significant predictor of the response to Question 3 (How successful do members of your family and close friends think your cochlear implant is?). Change in *Negative Tinnitus,* but not change in *Speech,* was a significant predictor of the response to Question 4 (If you knew that someone else in your family or a close friend had a similar condition to yours, would you encourage them to get a similar cochlear implant?).

A possible limitation of these analyses is that few participants gave responses other than the most favourable response. To explore further, we created four aggregate binary measures. The first measure distinguished the 103 participants who gave the most favourable response to each of the four questions from the 44 participants who gave a less than maximally favourable response to one or more of the questions. This way of defining the measure is the same as summing the responses given by each participant to the four questions and then distinguishing participants whose sum equalled 20 (four responses, each with a value of 5) from those with sums of 19 and less. Thus, in this case, the positive and negative categories were distinguished at a cut-point of 19.5. We created three further binary variables by applying the cut-points at values of 18.5, 17.5, and 16.5.

We then conducted a binary logistic regression analysis with each of the four new binary variables with *Standardised Speech* and *Standardised Negative Tinnitus* as the predictors. Figure OR3.2 plots the values of the beta coefficients and their BCa 95% c.i.s. *Standardised Negative Tinnitus* was not a significant predictor at any cut-point. *Standardised Speech* was a significant predictor at cut-points of 19.5 and 18.5. At a cut-point of 19.5, which achieved the most even (though still imperfect) balance between the numbers of participants in the positive and negative categories, *Standardized Speech* was a significantly more accurate predictor than was *Standardized Negative Tinnitus.*

Considered overall, the results of these analyses indicate that the change in speech reception was a stronger driver of satisfaction with implantation than was the change in annoyance due to tinnitus. That result, in turn, is compatible with the idea that improvement in speech reception is the primary outcome from implantation while improvement in tinnitus is a secondary outcome.

Figure OR3.1: Beta coefficients from binary logistic regression analyses for *Standardised Speech* (grey bars) and *Standardised Negative Tinnitus* (white bars) as predictors of participants giving either the most favourable response or any other response to each of four questions (Q1-4) about satisfaction with implantation. Error bars plot BCa 95% c.i.s. Pairs of numbers in parentheses are the numbers of participants in the positive (N +ve) and negative (N –ve) groups.

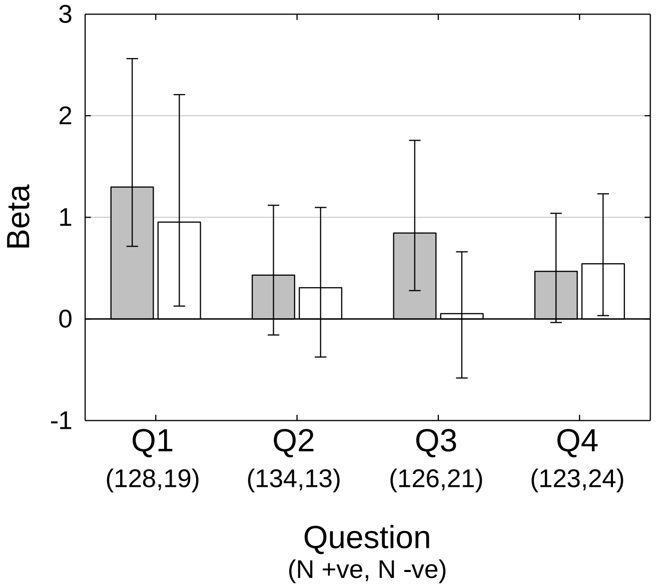

Figure OR3.2: Beta coefficients from binary logistic regression analyses for *Standardised Speech* (filled circles) and *Standardised Negative Tinnitus* (open circles) as predictors of binary variables created by applying four cut-points to the summed responses to the four questions about satisfaction with implantation. Error bars plot BCa 95% c.i.s. Pairs of numbers in parentheses are the numbers of participants in the positive (N +ve) and negative (N –ve) groups.

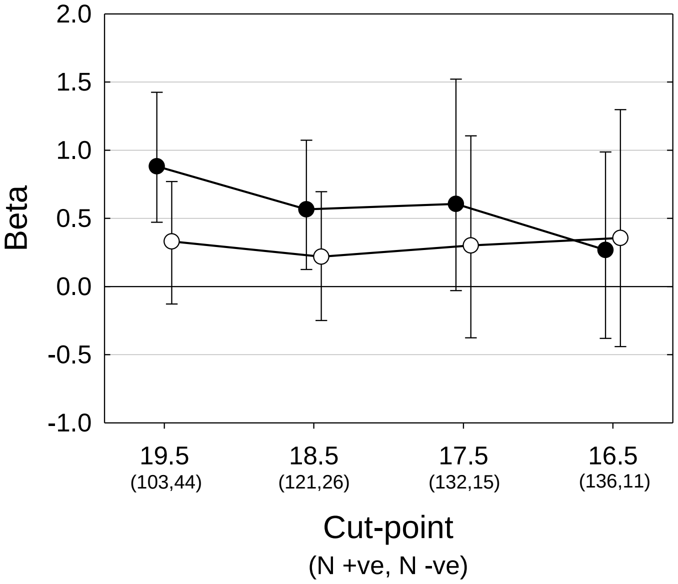

Online Resource 4: Multiple regression analyses

The three tables below report the results of multiple regression analyses in which *Speech* and *Tinnitus* were entered as potential predictors of each measure of HRQL. Table OR4.1 reports analyses of pre-operative measures. Table OR4.2 reports analyses of post-operative measures. Table OR4.3 reports analyses of the change in measures (post-operative minus pre-operative). Each table includes beta parameters (β) with 95% confidence intervals computed by bootstrapping (10,000 samples per analysis, bias-corrected and accelerated) (BCa 95% c.i.), levels of significance (Sig.), and adjusted R-squared (R^2). Entries are emboldened which explain a significant proportion of the variance. In addition to the five measures of HRQL which are described in the paper, the tables include analyses for the Glasgow Health Status Index (GHSI) and (in Table OR4.3 only) for the Glasgow Benefit Inventory. These two outcome measures are described in Online Resource 6.

The pattern of significance of *Speech* and *Tinnitus* as predictors here largely echoes the pattern of significance of their first-order correlations with the measures of HRQL which are reported in Table 4 in the paper. *Tinnitus* was a significant predictor of each measure of HRQL both before and after implantation. Change in *Tinnitus* was a significant predictor of the change in *EQ-5D-3L* and *EQ-VAS. Speech* was not a significant predictor pre-operatively where the majority of values of *Speech* were zero. *Speech* was a significant predictor of the majority of the measures of HQRL post-operatively. Change in *Speech* was a significant predictor of change only in *HUI2* and *HUI3.*

Both *Speech* and *Tinnitus* were significant predictors of *GHSI* both before and after implantation. Change in *Speech* and change in *Tinnitus* were significant predictors of the change in *GHSI* and of *GBI* which is a direct measure of change in health status.

Table OR4.1 Analyses of pre-operative data.

	Intercept				*Speech*				*Tinnitus*				R^2
Measure of HRQL	β	Sig.	BCa 95% c.i.		β	Sig.	BCa 95% c.i.		β	Sig.	BCa 95% c.i.		
			Lower	Upper			Lower	Upper			Lower	Upper	
EQ-5D-3L	**.866**	**p<.001**	**.809**	**.916**	-.001	n.s.	-.005	.003	**-.003**	**P=.001**	**-.005**	**-.001**	**.070**
EQ-VAS	**83.05**	**p<.001**	**78.96**	**87.00**	.000	n.s.	-.323	.297	**-.267**	**p<.001**	**-.366**	**-.156**	**.104**
HUI2	**.706**	**p<.001**	**.663**	**.749**	.000	n.s.	-.004	.005	**-.003**	**p<.001**	**-.004**	**-.001**	**.093**
HUI3	**.517**	**p<.05**	**.469**	**.566**	.001	n.s.	-.002	.004	**-.004**	**p<.001**	**-.005**	**-.002**	**.148**
SF-6D	**.814**	**p<.001**	**.783**	**.845**	.001	n.s.	-.002	.004	**-.002**	**p<.001**	**-.003**	**-.001**	**.136**
GHSI	**44.28**	**P<.001**	**40.93**	**47.70**	**-.216**	**p<.05**	**-.398**	**-.043**	**-.139**	**p<.01**	**-.227**	**-.043**	**.060**

Table OR4.2 Analyses of post-operative data.

Measure of HRQL	Intercept				*Speech*				*Tinnitus*				R^2
	β	Sig.	BCa 95% c.i.		β	Sig.	BCa 95% c.i.		β	Sig.	BCa 95% c.i.		
			Lower	Upper			Lower	Upper			Lower	Upper	
EQ-5D-3L	**.819**	**p<.001**	**.745**	**.894**	**.001**	**p<.05**	**.000**	**.002**	**-.003**	**p<.01**	**-.005**	**-.001**	**.095**
EQ-VAS	**81.06**	**p<.001**	**75.90**	**85.86**	.017	n.s.	-.052	.086	**-.245**	**p<.001**	**-.344**	**-.129**	**.101**
HUI2	**.742**	**p<.001**	**.655**	**.823**	**.002**	**p<.01**	**.001**	**.003**	**-.004**	**p<.001**	**-.005**	**-.002**	**.184**
HUI3	**.580**	**p<.001**	**.498**	**.656**	**.002**	**p<.001**	**.001**	**.003**	**-.003**	**p<.001**	**-.004**	**-.002**	**.174**
SF-6D	**.775**	**p<.001**	**.727**	**.824**	**.001**	**p<.05**	**.000**	**.001**	**-.002**	**p<.001**	**-.003**	**-.002**	**.187**
GHSI	**57.50**	**p<.001**	**52.76**	**62.15**	**.095**	**p<.01**	**.030**	**.160**	**-.311**	**p<.001**	**-.414**	**-.197**	**.211**

Table OR4.3 Analyses of change in measures.

Measure of HRQL	Intercept				*Speech*				*Tinnitus*				R^2
	β	Sig.	BCa 95% c.i.		β	Sig.	BCa 95% c.i.		β	Sig.	BCa 95% c.i.		
			Lower	Upper			Lower	Upper			Lower	Upper	
EQ-5D-3L	.0066	n.s.	-.0548	.0692	.0003	n.s.	-.0007	.0013	**-.0023**	**p<.05**	**-.0044**	**-.0003**	**.060**
EQ-VAS	.9147	n.s.	-2.989	4.833	-.0147	n.s.	-.0822	.0520	**-.1557**	**p<.05**	**-.3035**	**-.0215**	**.048**
HUI2	.0410	n.s.	-.0250	.1079	**.0016**	**p=.001**	**.0007**	**.0026**	-.0013	n.s.	-.0027	.0003	.098
HUI3	**.1061**	**p=.001**	**.0455**	**.1716**	**.0016**	**p=.001**	**.0007**	**.0024**	-.0011	n.s.	-.0025	.0004	.092
SF-6D	-.0068	n.s.	-.0379	.0235	.0002	n.s.	-.0003	.0008	-.0008	n.s.	-.0018	.0001	.026
GHSI	**9.604**	**p<.001**	**6.076**	**13.15**	**.1355**	**p<.001**	**.0741**	**.1983**	**-.1082**	**p<.05**	**-.2099**	**-.0055**	**.120**
GBI	**29.25**	**p<.001**	**24.32**	**34.67**	**.2556**	**p<.001**	**.1700**	**.3382**	**-.1881**	**P<.05**	**-.3426**	**-.0304**	**.206**

Online Resource 5: Analyses of cost effectiveness

Summary

This online resource reports estimates of the incremental cost-effectiveness ratio (ICER) of unilateral cochlear implantation for post-lingually deafened adults. The comparator was non-surgical intervention (i.e. the provision of high-powered acoustic hearing aids). Estimates of ICERs were derived by combining the gain in health utility measured with each of the four GPMs with the output of a decision-analytic model. The model was developed by the Peninsula Technology Assessment Group of the Universities of Exeter and Plymouth under a commission from the NHS R&D HTA programme on behalf of NICE. The model is described in Chapters 6 and 7 of Bond et al. (2009).

The analyses reported in this online resource show that the number of quality-adjusted life years (QALYs) estimated to be gained from implantation, and the resulting ICERs, vary widely depending on which GPM informs the analysis. The estimated number of QALYs gained ranges from .14 (SF-6D), to .48 (EQ-5D-3L), 1.64 (HUI2), and 2.40 (HUI3). The estimated ICERs range from £241,357/QALY (SF-6D), to £70,411/QALY (EQ-5D-3L), £20,690/QALY (HUI2), and £14,163/QALY (HUI3).

In the UK, treatments which gain QALYs for less than about £20,000 are unlikely to be rejected on grounds of cost-ineffectiveness, while special reasons are required to adopt treatments that gain QALYs for more than about £30,000 (Rawlins & Culyer, 2004). By those criteria, unilateral implantation of post-lingually deafened adults would be judged to represent acceptable value for money when analyses were informed by HUI3 and HUI2, but not when analyses were informed by EQ-5D-3L or SF-6D.

Orientation

The first section below describes the decision-analytic model. The second section describes how we used the model and then reports the results which we obtained.

Decision analytic model of Bond et al. (2009)

The model describes the pathways – and the associated probability with which each pathway will be taken and the associated cost of taking it – which a patient can follow after referral for implantation. Montecarlo simulations were run which traced the pathways followed by cohorts of 1000 patients. The cost of managing each simulated patient and the number of quality-adjusted life years (QALYs) gained by each simulated patient were estimated, and the average cost and average gain in QALYs were calculated across the cohort. Those values were compared with corresponding values estimated for non-surgical intervention. By comparing the two sets of estimates, the incremental cost and incremental benefit of providing implantation could be estimated and an ICER could be calculated.

The following four paragraphs describe features of cochlear implantation that informed the structure of the model. The fifth paragraph describes the model itself.

Background: Cochlear implant

A cochlear implant consists of two main parts: an external part and an internal part. The external part consists of a microphone and sound processor which are typically combined in a device that looks like an acoustic hearing aid and is worn behind the ear. This package connects to a transmitter coil. The processor converts sounds detected by the microphone into data. The data, along with electrical power, are sent by the transmitter coil as radio-frequency signals through the skin of the scalp to the internal part. The internal part consists of a receiver coil which detects the power and data transmitted by the transmitter coil, a microprocessor which converts the data into electrical impulses which are delivered to electrodes that are implanted surgically in the coiled structure of the inner ear (the cochlea). The receiver coil and microprocessor are placed in a well

which is fashioned surgically in the mastoid bone behind the implanted ear. The transmitter and receiver coils are held in alignment across the scalp by opposing magnets.

Background: Care pathway for cochlear implantation

The care pathway for cochlear implantation in the UK starts with referral to a specialised centre for assessment of candidacy by audiometric testing and CT and MR imaging. Criteria for implantation as an adult are: a severe-profound hearing impairment in both ears which developed after the acquisition of spoken language; a patent auditory nerve in at least one ear; inability to identify correctly more than 50% of the content words in pre-recorded sentences without lipreading; the physical robustness to withstand a general anaesthetic and then to attend outpatient appointments; the intellectual capacity to participate in post-operative tuning of the implanted device. Implantation requires a general anaesthetic and a brief in-patient stay. About a month after the operation, the patient makes an outpatient visit for the implant to be activated and the levels of stimulation on each electrode adjusted to give audible pain-free sensations. Further outpatient visits may follow for the implant to be adjusted and for faults to be diagnosed and corrected.

Background: Use of acoustic hearing aids

Where a patient has used an acoustic hearing aid in one or both ears prior to implantation, they may continue to use an aid in one ear after implantation if the patient and their clinical advisors decide that implantation would be clinically effective in the other ear.

Background: Medical / surgical complications

The majority of cases are implantation straightforward. Medical / surgical complications may involve the surgical wound, or a failure of parts of the implant system. Problems with the wound are generally resolved by revision surgery and antibiotic regimes. Failed internal parts can are generally removed and replaced surgically without loss of function. External parts can fail and so require replacement and are generally upgraded routinely every six-to-ten years.

Decision-analytic model

The decision-analytic model is a two-level state-transition (Markov) model which describes the events, and their associated probabilities and costs, that can occur to a patient referred for implantation. The model is illustrated in Figure OR5.1, below, which has been redrawn from Figures 5 and 6 of Bond et al. (2009, Page 85). The upper level describes the main pathways followed by patients. The lower level describes the clinical and device-related events that can occur to users of a unilateral implant. Bond et al. (2009) obtained the probabilities of events and their associated costs from published sources, supplemented in a few instances by expert opinion. The probabilities are listed in Table OR5.1, the costs in Table OR5.2, and the base-case gain in health utility in Table OR5.3.

The alternative to implantation was assumed to entail only the replacement of an acoustic hearing aid every 5 years. The model estimated the average cost of this alternative so that the incremental cost of providing a unilateral implant could be estimated.

For each alternative, a cohort of 1000 modelled patients was followed from age 50 until death. Transitions between states could occur every six months. Table OR5.4 contains the main results reported by Bond et al. (2009) in terms of the gain in quality-adjusted life years (QALYs) and the associated incremental cost-effectiveness ratio (ICER) estimated by the model. For a patient referred at age 50 years, the model estimated that the incremental costs over the patient's remaining life time averaged £33,959 gaining 2.40 incremental QALYs and yielding an ICER of £14,163/QALY (Table 75 in Bond et al., 2009).

Our use of the output of the decision-analytic model of Bond et al. (2009)

Bond et al. (2009) summarised results in a graph showing the relationship between the incremental gain in health utility (ΔU) and Incremental Net Benefit (INB) (Figure 37 in Bond et al., 2009). INB is the difference between the value of the additional QALYs gained from an intervention and the incremental cost of gaining them. NICE requires analysts to value QALYs maximally at £30,000 (NICE, 2013). Thus, INB = £30,000 x ΔQ - ΔC, where ΔQ is the additional QALYs gained and ΔC is the incremental cost of gaining them. Bond et al. (2009) estimated that the relationship between INB and ΔU was linear. It can be deduced from their Figure 37 to correspond to the equation:

$$\text{INB} = £366{,}000 \times \Delta U - £34{,}130 \qquad \text{Equation 1}$$

By substitution and rearrangement, the QALYs gained can be calculated for any value of ΔU as:

$$\Delta Q = (366{,}000 \times \Delta U - 171)/30{,}000 \qquad \text{Equation 2}$$

The ICER can be calculated as:

$$\text{ICER} = £(33{,}959 \times 30{,}000)/(366{,}000 \times \Delta U - 171) \qquad \text{Equation 3}$$

We used Equations 2 and 3 to calculate the QALYs that would be gained and the resulting ICER if the incremental gain in utility was that estimated with each GPM.

The results are listed in Table OR5.5 and are illustrated graphically in Figure OR5.2 which shows the relationship between ΔU and INB. The diagonal line sloping upwards from bottom left to top right plots Equation 1. The filled circles on the line show the relationship between ΔU and INB for each GPM. The values in pounds on the right of the graph are the values of INB estimated with each GPM. They range from -£29,738 (SF-6D), to -£19,490 (EQ-5D-3L), £15,280 (HUI2), and £37,972 (HUI3). The numbers of QALYs gained ranged from .14 (SF-6D), to .48 (EQ-5D-3L), 1.64 (HUI2), and 2.40 (HUI3). The ICERs ranged from £241,357/QALY (SF-6D), to £70,411/QALY (EQ-5D-3L), £20,690/QALY (HUI2), and £14,163/QALY (HUI3).

In the UK, treatments which gain QALYs for less than about £20,000 are unlikely to be rejected on grounds of cost-ineffectiveness, while special reasons are required for adopting treatments that gain QALYs for more than about £30,000 (Rawlins & Culyer, 2004). By those criteria, unilateral implantation of post-lingually deafened adults would be judged to represent acceptable value for money when analyses were informed by HUI3 and HUI2, but not when analyses were informed by EQ-5D-3L or SF-6D.

Reflection

The results create a dilemma insofar as the GPM preferred by NICE, EQ-5D-3L, indicates that unilateral cochlear implantation of adults is not a good use of resources, while an alternative GPM, HUI3, indicates that implantation is a good use of resources. NICE resolved the dilemma by basing policy on analyses informed by HUI3 (NICE, 2009). That judgement, in turn, implicitly acknowledged that EQ-5D-3L failed to capture all of the benefits of a treatment that improved hearing.

Explanation and cross-check

Bond et al. (2009) obtained an estimate of the gain in health utility associated with implantation from UK Cochlear Implant Study Group (2004). That study reported results from 311 patients using HUI3. Of them, a subset of 147 patients provided the data reported in the present paper. By chance, the average gain in utility measured with HUI3 for the 311 patients was the same as the average gain measured for the 147 patients. That is why the base-case assessed by Bond et al. (2009) involved the same gain in utility, .197, as the gain reported for HUI3 in the present paper.

That equality allows a check on the calculations that we used to estimate the QALYs gained and ICERs. The numbers of QALYs gained and the ICER for HUI3 in Table OR5.5 were obtained by substituting .197 for ΔU in Equations 2 and 3. They are the same as the numbers of QALYs gained and the ICER reported by Bond et al. (2009) which are included in Table OR5.4.

References

Bond M, Mealing S, Anderson R, Elston J, Weiner G, Taylor RS, Hoyle M, Liu Z, Proce A, Stein K. (2009). The effectiveness and cost-effectiveness of cochlear implants for severe to profound deafness in children and adults: a systematic review and economic model. *Health Technology Assessment*, Vol. 13, No. 44. (https://www.ncbi.nlm.nih.gov/pubmed/19799825)

National Institute for Health and Care Excellence. (2013). *Guide to the Methods of Technology Appraisal 2013*. Retrieved 30th November 2017, from https://www.nice.org.uk/process/pmg9/chapter/foreword

Rawlins, M.D., Culyer, A.J. (2004). National Institute for Clinical Excellence and its value judgements. *British Medical Journal* 329: 224-227.

UK Cochlear Implant Study Group. (2004). Criteria of candidacy for unilateral cochlear implantation in postlingually deafened adults II: Cost-effectiveness analysis. *Ear and Hearing* 25: 336-360.

Figure OR5.1 The two-level state-transition model of Bond et al. (2009)

Upper level

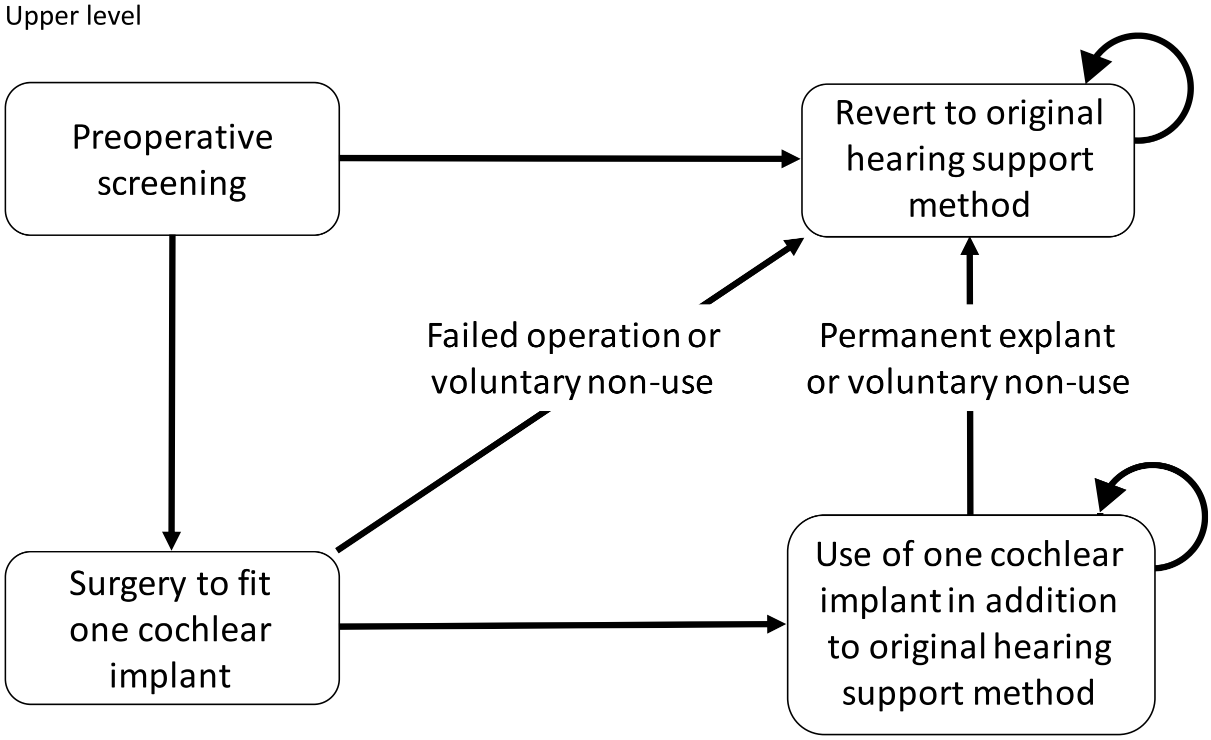

Lower level

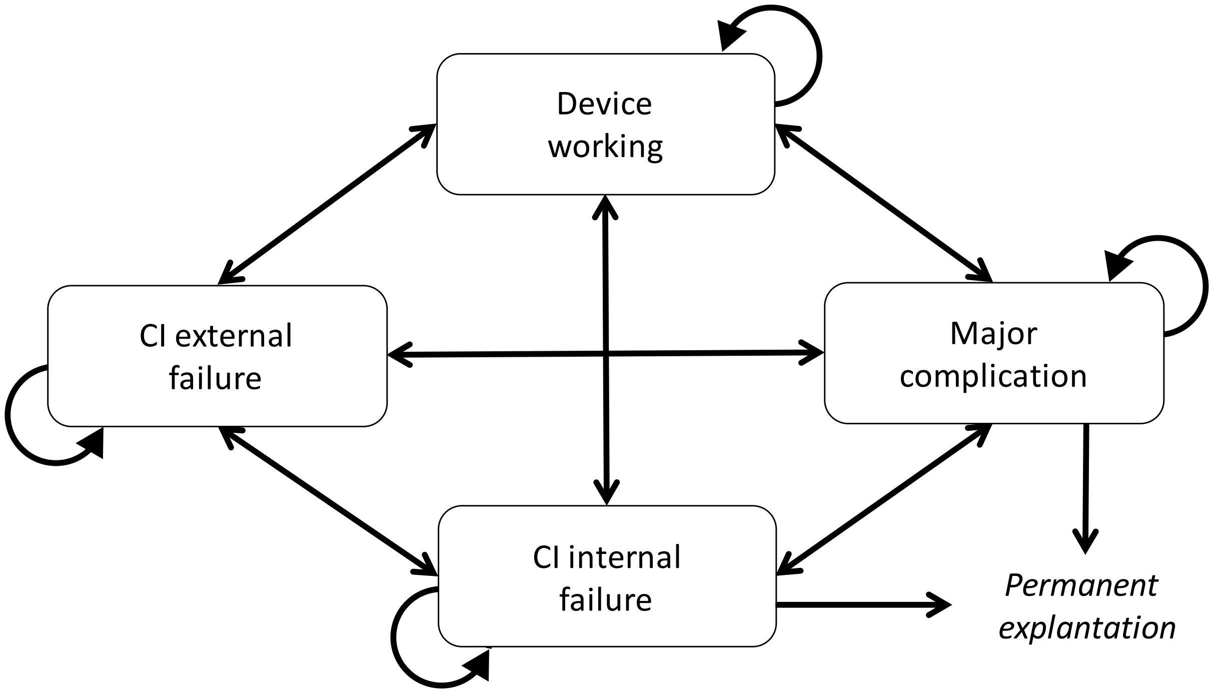

Figure OR5.2 Relationship between utility gain and incremental net benefit (redrawn from Figure 37 in Bond et al., 2009)

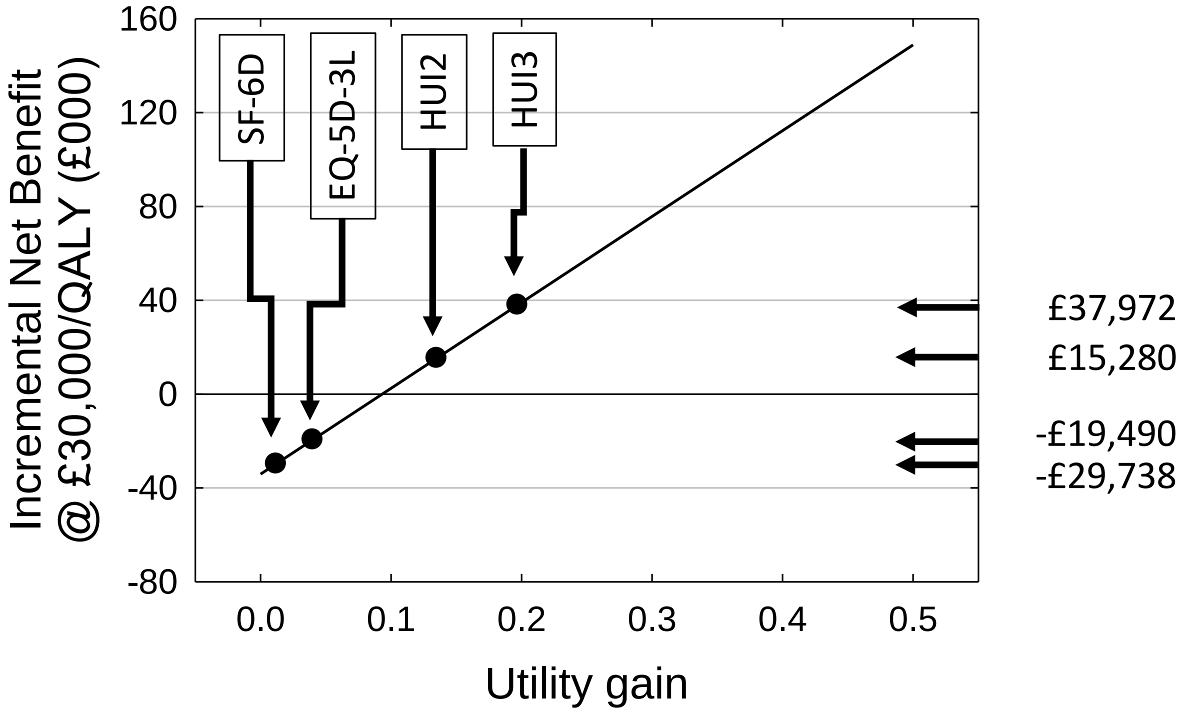

Table OR5.1 Model Parameters taken from Table 50 of Bond et al. (2009, Page 94).

Parameter	Base-case value
Time horizon	Lifetime
Annual discount rate	3.5%
Starting age	50 years
Gender distribution	Male: 41%, Female: 59%
Percentage of candidates who gain some benefit from acoustic hearing aids	50%
Percentage of unilateral implantees who use an acoustic hearing aid in their other ear	70%
Percentage of referrals who do not proceed to implantation	30%
Probability of surgical death	0.0
General mortality	Age dependent in accordance with UK Government Actuary's life tables
Percentage of operations which are abandoned	0%
Average lifetime of an acoustic hearing aid	5 years
6-month probability of failure of external components	0.062
6-month probability of failure of internal components	Time-dependent function based on empirical survival data corresponding, approximately, to a linear decline in cumulative survival to 96.5% after 40 years. (Figure 8 in Bond et al. (2009).)
General mortality	Age dependent in accordance with UK Government Actuary's life tables
6-month probability of major medical / surgical complication	Year 1: 0.02, Year 2+: 0.002
Probability of voluntary permanent non-use of implant	2.36% after 2 years. Full compliance before and after that point.
Probability of non-reimplantation of internal component during any surgical procedure	0.115
Percentage of internal component failures during warranty period	0.7%

Table OR5.2 Cost parameters taken from Table 53 of Bond et al. (2009, Page 99).

Parameter	Value (2006 £)
Pre-surgical assessment of candidacy	4,011
Unilateral implantation (excluding implant system)	2,814
Unilateral implant system	14,661
Replacement of digital hearing aid	100
Tuning and maintenance in Year 1	5,000
Maintenance in Year 2	798
Maintenance in Year 3	756
Maintenance in year 4+	596
10-yearly upgrade of external part	4,114
Major complication	7,777
Internal component failure during warranty	2,814
Internal component failure after warranty	17,425
External component failure during warranty	0
External component failure after warranty	4,114

Table OR5.3 Base-case utility parameter taken from Table 59 of Bond et al. (2009, Page 106).

Estimated gain in utility	0.197 (95% confidence interval 0.176 to 0.218)

Table OR5.4 Base-case results taken from Table 75 of Bond et al. (2009, Page 127).

	Costs (2006 £)	QALYs	Incremental costs	Incremental QALYs	ICER (£/QALY)
Non-cochlear implant use	248	8.20	-	-	-
Unilateral cochlear implant use	34,207	10.60	33,959	2.40	14,163

Table OR5.5 Results of the current analyses

	GPM			
	EQ-5D-3L	HUI2	HUI3	SF-6D
Gain in health utility	.040	.135	.197	.012
Gain in QALYs	.48	1.64	2.40	.14
INB[a]	-£19,490	£15,280	£37,972	-£29,738
ICER[b]	£70,411/QALY	£20,690/QALY	£14,163/QALY	£241,357/QALY

[a] Incremental Net benefit with QALYs valued at £30,000

[b] Incremental Cost-effectiveness Ratio

Online Resource 6: Relationship of *Speech* and *Tinnitus* to physical health and psycho-social well-being

We sought further understanding of the different ways in which speech reception and tinnitus influence HRQL by administering two additional questionnaires: the Glasgow Benefit Inventory (GBI) and the Glasgow Health Status Index (GHSI) (Robinson, Gatehouse & Browning, 1996). We first describe the two questionnaires. We then report an analysis which indicates that participants perceived tinnitus to be more strongly related to physical health, while speech reception was more strongly related to psycho-social well-being.

Glasgow Benefit Inventory (GBI)

The GBI is a domain-specific self-report measure of *change* in health status. It is composed of 18 questions selected to be relevant to otorhinolarygological disorders. The GBI provides a direct measure of the change in aspects of health and quality of life associated with an event, such as the onset of impairment or an intervention intended to alleviate it. With the event specified as cochlear implantation, questions take the form: "Since you received your cochlear implant, do you have more or less self-confidence?". Responses are made on 5-point Likert scales that are labelled appropriately for each question. For the question, above, the labels are: "Much more self-confidence", "More self-confidence", "No change", "Less self-confidence", and "Much less self-confidence". Each response is scored with an integer in the range from -2 to +2, where -2 is assigned to the change with the greatest negative impact (e.g. "Much less self-confidence") and +2 to the change with the greatest positive impact (e.g. "Much more self-confidence"). The 18 scores are averaged and the average is re-scaled to an overall score in the range from -100 to +100 with the formula:

Overall Score = Average x 50

Robinson, Gatehouse, and Browning reported that each of the 18 questions loaded on one of three factors which they labelled *Psycho-social health (PS)*, *Social support (SS)*, and *Physical health (PH)*. Table OR6.1 relates to the GBI. Its columns contain:

1. The number of the question.
2. Which factor the question loads on (PS, SS, or PH).
3. The wording of the question.
4. The label of the first point on the Likert Scale for that question.
5. The label of the last point on the Likert Scale for that question.
6. The mean value of the overall score for the question.
7. The standard deviation of the mean value.
8. The rank of the mean value such that the largest positive change was assigned the rank of 1.

Table OR6.1: Glasgow Benefit Inventory (GBI).

No.	Factor	GBI Question	Likert point 1	Likert point 5	Score Mean	Score SD	Score Rank
1	PS	Has getting a cochlear implant affected the things you do?	Much worse	Much better	82.99	31.78	2
2	PS	Has getting a cochlear implant made your overall life better or worse?	Much worse	Much better	88.44	27.49	1
3	PS	Since you received your cochlear implant, have you felt more or less optimistic about the future?	Much less optimistic	Much more optimistic	73.13	33.24	3
4	PS	Since you received your cochlear implant, do you feel more or less embarrassed when with a group of people?	Much more embarrassed	Much less embarrassed	59.18	43.01	7
5	PS	Since you received your cochlear implant, do you have more or less self-confidence?	Much less self-confidence	Much more self-confidence	61.22	42.12	5
6	PS	Since you received your cochlear implant, have you found it easier or harder to deal with company?	Much harder	Much easier	66.33	35.68	4
7	SS	Since you received your cochlear implant, do you feel that you have more or less support from your friends?	Much less support	Much more support	36.73	47.66	10
8	PH	Since you received your cochlear implant, have you been to your family doctor for any reason, more or less often?	Much more often	Much less often	11.56	34.65	16
9	PS	Since you received your cochlear implant, do you feel more or less confident about job opportunities?	Much less confident	Much more confident	34.01	37.47	12
10	PS	Since you received your cochlear implant, do you feel more or less self-conscious?	Much more self-conscious	Much less self-conscious	30.95	52.45	13
11	SS	Since you received your cochlear implant, are there more or fewer people who really care about you?	Many fewer people	Many more people	14.97	33.83	15
12	PH	Since you received your cochlear implant, do you catch colds or infections more or less often?	Much more often	Much less often	5.78	29.56	17
13	PH	Since you received your cochlear implant, have you had to take more or less medicine for any reason?	Much more medicine	Much less medicine	4.08	30.13	18
14	PS	Since you received your cochlear implant, do you feel better or worse about yourself?	Much worse	Much better	61.22	39.17	6
15	SS	Since you received your cochlear implant, do you feel that you have had more or less support from your family?	Much less support	Much more support	28.91	40.51	14
16	PS	Since you received your cochlear implant, are you more or less inconvenienced by your hearing problem?	Much more inconvenienced	Much less inconvenienced	57.14	42.60	8
17	PS	Since you received your cochlear implant, have you been able to participate in more or fewer social activities?	Many fewer activities	Many more activities	35.03	35.80	11
18	PS	Since you received your cochlear implant, have you been more or less inclined to withdraw from social situations?	Much more inclined	Much less inclined	40.82	44.19	9

The Glasgow Health Status Inventory (GHSI)

The GHSI is a domain-specific self-report measure of health *status*. It is composed of 18 items selected to be relevant to disorders of hearing; for example: "Is your self-confidence affected by any problem with your hearing?". Responses are made on 5-point Likert scales that are labelled appropriately for each question. For the question above, the labels are: "Not at all affected", "Very slightly affected", "Slightly affected", "Moderately affected", and "Greatly affected". Each response is scored with an integer in the range 1 to 5, where 1 is assigned to the most negative response and 5 to the most positive response. The average of the 18 scores is re-scaled to an overall score in the range from 0 to 100 with the formula:

Overall Score = (Average-1) x 25.

The GHSI is a partner to the GBI in the sense that each question in the GHSI has a corresponding question in the GBI. Table OR6.2 relates to the GHSI. Its columns contain:

1. The number of the question.
2. Which factor the question loads on (PS, SS, or PH).
3. The wording of the question as used in the study.
4. The label of the first point on the Likert Scale for that question.
5. The label of the last point on the Likert Scale for that question.
6. The mean value of the overall score measured before participants received cochlear implants (Pre-op).
7. The ranks of those scores such that the lowest score was assigned the rank of 1.
8. The mean value of the overall score measured 9 months after participants had received cochlear implants (Post-op).
9. The change in score assigned to a question (Post-op score minus Pre-op score).
10. The ranks of the changes in score such that largest positive change was assigned the rank of 1.

Relationship of Factor Scores to *Speech* and *Tinnitus*

Three factor scores were calculated for each participant by averaging responses to the appropriate sub-set of questions in the GBI and by subtracting pre- from post-operative scores for the corresponding questions in the GHSI. Table OR6.3 reports Kendall rank correlation coefficients between the factor scores and the measures of change in *Speech* and *Tinnitus.* Change in *Speech* was correlated significantly with the psycho-social well-being factor, but with neither of the other two factors, while change in *Tinnitus* was correlated significantly with the physical-health factor, but with neither of the other two factors. Thus, this analysis provides additional evidence that tinnitus was perceived by participants to be more strongly related to conventional construals of 'health' than was speech reception.

Table OR6.2: Glasgow Health Status Inventory (GHSI).

No.	Factor	GHSI Question	Likert point 1	Likert point 5	Pre-op	Rank	Post-op	Score Change	SD	Rank
1	PS	How often does any problem with your hearing affect the things you do?	Frequently or all of the time	Never	13.10	2	43.37	30.27	30.30	2
2	PS	How much does any problem with your hearing affect your overall life?	Not at all affected	Greatly affected	14.63	3	44.05	29.42	30.79	3
3	PS	Which of the following statements best describes your view of the future?	Pessimistic	Optimistic	69.73	15	79.59	9.86	30.63	11
4	PS	As a result of any problem with your hearing, how often do you feel embarrassment when with a group of people?	Frequently or all of the time	Never	21.26	5	54.93	33.67	31.47	1
5	PS	Is your self-confidence affected by any problem with your hearing?	Not at all affected	Greatly affected	30.44	7	57.99	27.55	34.46	5
6	PS	How often does any problem with your hearing affect how you deal with company?	Frequently or all of the time	Never	18.71	4	48.13	29.42	29.65	4
7	SS	How much support do you have from your friends?	Little or no support	A great deal of support	70.58	16	74.66	4.08	30.83	14
8	PH	How often do you consult your family doctor for any reason?	Seven or more times a year	Never	54.25	14	56.97	2.72	20.09	15
9	PS	How often does any problem with your hearing affect how confident you are about job opportunities?	Frequently or all of the time	Never	34.69	9	56.97	22.28	41.19	10
10	PS	How often does any problem with your hearing make you feel self-conscious?	Frequently or all of the time	Never	27.89	6	54.93	27.04	29.19	6
11	SS	How many people really care about you?	None	More than six people	90.14	18	89.97	-0.17	16.68	18
12	PH	If there is a cold or infection going around, how often do you usually catch it?	Frequently or all of the time	Never	49.83	13	54.08	4.25	23.11	13
13	PH	How often do you have to take medicine for any reason?	Frequently or all of the time	Never	42.35	12	42.69	0.34	26.97	17
14	PS	Does any problem with your hearing affect the way you feel about yourself?	Feelings greatly affected	Feelings never affected	37.59	11	63.10	25.51	34.18	7
15	SS	How much support do you have from your family?	Little or no support	A great deal of support	80.78	17	82.82	2.04	19.08	16
16	PS	How often are you inconvenienced by any problem with your hearing?	Daily	Never	9.01	1	32.99	23.98	31.22	9
17	PS	How often do you participate in social activities?	Less than 3 times a month	More than 3 times a day	35.20	10	43.71	8.50	35.17	12

18	PS	How often do you feel inclined to withdraw from social situations?	Frequently or all of the time	Never	30.61	8	55.78	25.17	30.90	8

Table OR6.3 Factor scores from GBI and GHSI and their coefficients of correlation with change in *Speech* and *Tinnitus.* Factor scores were derived directly from subsets of the questions in the GBI and by subtracting pre-operative scores from post-operative scores for corresponding questions in the GHSI. The significance (Sig.) of Kendal rank correlation coefficients (τ) was assessed against Bonferroni-adjusted alpha levels of .0167 (.05/3) (*) and .0033 (.01/3) (**). (n.s. = not significant.)

Questionnaire	Factor	Factor score		Correlation with change in *Speech*		Correlation with change in *Tinnitus*	
		Mean	SD	τ	Sig.	τ	Sig.
	Psycho-social	57.54	26.38	.395	**	-.070	n.s.
GBI	Social-support	26.87	34.09	.029	n.s.	-.068	n.s.
	Physical-health	7.14	23.49	-.095	n.s.	-.161	*
	Psycho-social	24.39	19.75	.213	**	-.092	n.s.
GHSI	Social-support	1.98	16.01	.100	n.s.	.039	n.s.
	Physical-health	2.44	16.27	.017	n.s.	-.195	**

Reference

Robinson, K., Gatehouse, S., Browning, G.G. (1996). Measuring patient benefit from otorhinolaryngological surgery and therapy. Annals of Otology Rhinology and Laryngology 105: 415-422.
